# In vitro activity of tedizolid and linezolid against *Staphylococcus epidermidis* isolated from prosthetic joint infections

**DOI:** 10.1007/s10096-017-2966-z

**Published:** 2017-03-22

**Authors:** C. Littorin, B. Hellmark, Å. Nilsdotter-Augustinsson, B. Söderquist

**Affiliations:** 1 School of Medical Sciences, Faculty of Medicine and Health, Örebro University, Örebro, Sweden; 2 Department of Laboratory Medicine, Clinical Microbiology, Örebro University Hospital, Örebro, Sweden; 3 Division of Infectious Diseases, Department of Clinical and Experimental Medicine, Faculty of Health Sciences, Linköping University, Linköping, Sweden; 4 Department of Infectious Diseases, County Council of Östergötland, Linköping, Sweden; 5 Departments of Infectious Diseases and Laboratory Medicine, Clinical Microbiology, Örebro University Hospital, SE-701 85 Örebro, Sweden

## Abstract

Prosthetic joint infections (PJIs) are rare but long-lasting and are serious complications without any spontaneous resolution, requiring additional surgery and long-term treatment with antibiotics. Staphylococci are the most important aetiological agents of PJIs, and among the coagulase-negative staphylococci *Staphylococcus epidermidis* is the most common. However, *S. epidermidis* often displays multidrug resistance (MDR), demanding additional treatment options. The objective was to examine the effectiveness of tedizolid and linezolid against *S. epidermidis* isolated from PJIs. The standard antibiotic susceptibility pattern of *S. epidermidis* (*n* = 183) obtained from PJIs was determined by disc diffusion test, and MIC was determined by Etest for tedizolid, linezolid, and vancomycin. Tedizolid displayed MIC values ranging from 0.094 to 0.5 mg/L (MIC_50_: 0.19 mg/L, MIC_90_: 0.38 mg/L), linezolid MIC values ranging from 0.25 to 2 mg/L (MIC_50_: 0.75 mg/L, MIC_90_: 1 mg/L), and vancomycin MIC values ranging from 0.5 to 3 mg/L (MIC_50_ and MIC_90_ both 2 mg/L). According to the disc diffusion test, 153/183 (84%) isolates were resistant to ≥3 antibiotic groups, indicating MDR. In conclusion, *S. epidermidis* isolates from PJIs were fully susceptible, and the MIC_50_ and MIC_90_ values for tedizolid were two- to four-fold dilution steps lower compared with linezolid. Tedizolid is not approved, and there are no reports of long-term treatment, but it may display better tolerability and fewer adverse effects than linezolid; it thus could be a possible treatment option for PJIs, alone or in combination with rifampicin.

## Introduction

Joint replacements have significantly improved the quality of life for many patients. Prosthetic joint infections (PJIs) are rare complications, affecting less than 1% of primary joint replacement surgeries [1]. However, PJIs are long-lasting and serious conditions without any spontaneous resolution, causing high morbidity and mortality, extensive costs, and substantial suffering for the patient due to disability, pain, prolonged hospitalization, revision surgery, and long-term treatment with antibiotics [2–4].

Staphylococci are the most important aetiological agents of PJIs [2–4], and *S. epidermidis* is the most common of the CoNS. This species regularly forms a biofilm, and so the proposed treatment of choice for PJIs is rifampicin, which is efficient against staphylococci in biofilm [2, 4]. Rifampicin is always administered in combination with another antimicrobial agent such as fluoroquinolones, clindamycin, or fusidic acid in order to hinder the emergence of resistance [2–4]. However, *S. epidermidis* often displays a multidrug resistance (MDR) phenotype [5]. Linezolid could be one of the few remaining options for oral long-term treatment, but is bacteriostatic, not proven to eradicate staphylococci present in biofilm [6], and frequently exhibits adverse effects [7]. Nevertheless, it has been reported to successfully treat chronic osteomyelitis including PJIs [7, 8].

The aim of this study was to investigate the antibiotic activity of a new oxazolidinone, tedizolid, and to compare its MIC values with those of linezolid, among *S. epidermidis* isolated from PJIs.

## Materials and methods

*S. epidermidis* isolates (*n* = 183) were obtained during various surgical procedures due to suspected or verified PJI at the University Hospitals of Örebro and Linköping from 1999 to 2015 and from 1993 to 2015, respectively. The finding of *S. epidermidis* in multiple tissue samples (≥2) was interpreted as a PJI in accordance with the proposed definition of PJI [3, 4]. Isolates were collected from patients with infected hip (*n* = 126), knee (*n* = 41), shoulder (*n* = 12), or elbow (*n* = 4) joint prostheses, then identified to the species level according to routine laboratory procedures and confirmed by MALDI-TOF MS (MicroflexLT and Biotyper 3.1, Bruker Daltonics, Bremen, Germany).

Standard antibiotic susceptibility testing by disc diffusion test (DDT) and MIC determination was performed according to EUCAST guidelines. MIC was determined by Etest for tedizolid, linezolid (Liofilchem, Roseto degli Abruzzi, Italy), and vancomycin (BioMérieux, Marcy-l’Etoile, France). DDT was performed for cefoxitin (30 μg), fusidic acid (10 μg), erythromycin (15 μg), clindamycin (2 μg), trimethoprim/sulfamethoxazole (25 μg), gentamicin (10 μg), norfloxacin (10 μg), and rifampin (5 μg) (Oxoid, Basingstoke, Hampshire, England).

## Results

**Fig. 1:**
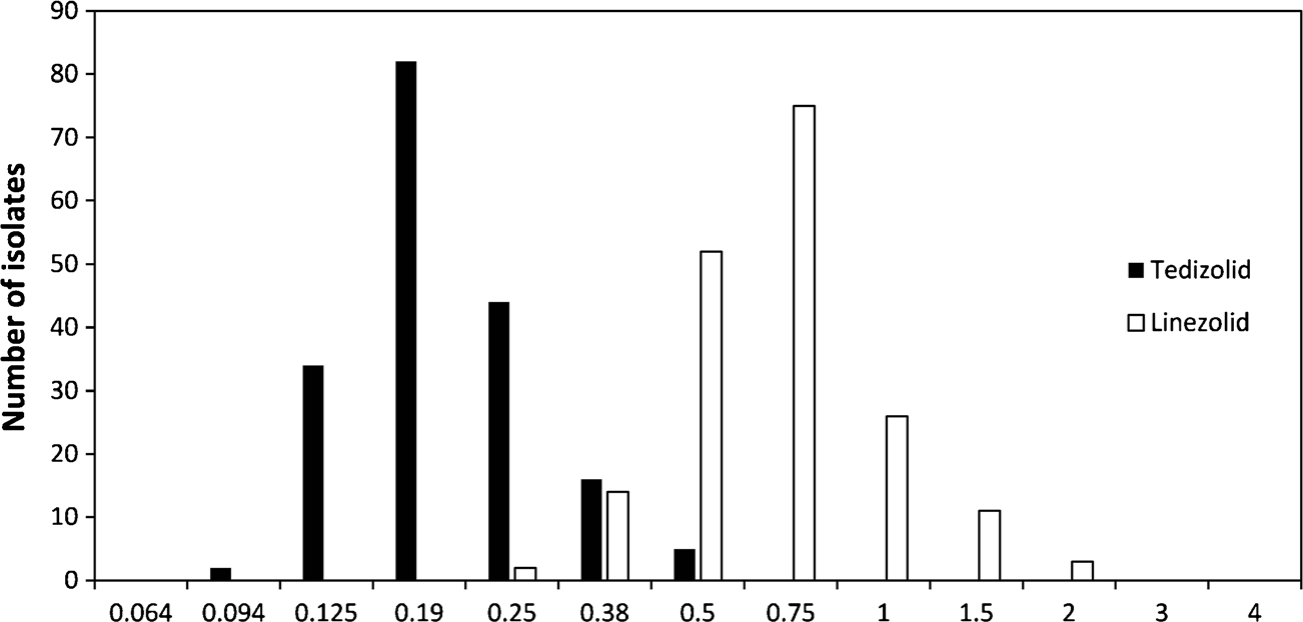
Distribution of the minimum inhibition concentrations (MICs) of tedizolid and linezolid, as determined with Etest, for 183 isolates of *Staphylococcus epidermidis* obtained from prosthetic joint infections

Results of the antimicrobial susceptibility testing of tedizolid and linezolid are shown in Fig. 1. Tedizolid displayed MIC values ranging from 0.094 to 0.5 mg/L (MIC_50_: 0.19 mg/L, MIC_90_: 0.38 mg/L) and linezolid MIC values ranging from 0.25 to 2 mg/L (MIC_50_: 0.75 mg/L, MIC_90_: 1 mg/L). All isolates were susceptible according to EUCAST breakpoints (≤0.5 mg/L and ≤4 mg/L, respectively). The MIC values of vancomycin ranged from 0.5 to 3 mg/L (MIC_50_ and MIC_90_ both 2 mg/L). The breakpoint for susceptibility according to EUCAST is ≤4 mg/L.

According to the DDT, 22% of the *S. epidermidis* isolates were susceptible to cefoxitin, 56% to fusidic acid, 42% to clindamycin, 37% to erythromycin, 72% to rifampicin, 28% to gentamicin, 19% to norfloxacin, and 27% to sulfamethoxazole-trimethoprim. In total, 153/183 (84%) isolates were resistant to ≥3 antibiotic groups, indicating MDR. There were no differences between isolates displaying MDR and non-MDR in MIC_50_ and MIC_90_, respectively, neither for tedizolid nor for linezolid.

## Discussion

This study investigated the antibiotic activity of a new oxazolidinone, tedizolid, compared to linezolid against *S. epidermidis* isolated from PJIs. All isolates were fully susceptible to both antibiotics according to EUCAST breakpoints. The MIC_50_ and MIC_90_ values for tedizolid were two- to four-fold dilution steps lower than the corresponding values for linezolid. Our results are in concordance with previous studies [9–14].

All *S. epidermidis* isolates were also susceptible to vancomycin according to the EUCAST breakpoint. However, 111/183 (61%) showed MIC values ≥2 mg/L, and thus an increased risk of nephrotoxicity may be present if the trough value of the plasma concentration is intended to be approximately 20 mg/L. Heterogeneous glycopeptide intermediate *S. epidermidis* (hGISE) may also be present among *S. epidermidis* isolated from PJIs [15], which will not be detected if only standard Etest methods are used. The present study also found a high percentage (84%) of MDR. The proportions of isolates susceptible to the key antimicrobial agents for oral long-term treatment of PJIs, rifampicin and fluoroquinolones, were 72% and 19%, respectively. In addition, an association between hGISE and MDR has been reported [15]. Subsequently, in some cases an oxazolidinone may be the only remaining antimicrobial agent for oral treatment of a stable, retained implant following debridement or after explantation when performing two-stage exchange revision surgery. Linezolid has been employed for treatment of these infections, including long-term therapy [7, 8]. Tedizolid, on the other hand, is approved for 6 days’ treatment of skin and soft tissue infections and there are no reports of long-term treatment or treatment of orthopaedic infections. In a phase I study [16], 30 healthy subjects received 200–1200 mg tedizolid once a day for 21 days, and three showed adverse events such as alteration of haematological parameters and elevation of liver enzymes. Tedizolid seems to be well-tolerated, and adverse events are less common among patients treated with tedizolid compared to linezolid [17]. The myelosuppressive effect of tedizolid seems to be lower than that of linezolid. Neither peripheral nor optic neuropathy were found in either patients following 10 days of treatment or rats after up to 9 months’ exposure to high doses of tedizolid [17]. Although tedizolid is a more potent inhibitor of mitochondrial protein synthesis than linezolid, mitochondrial recovery may be superior for tedizolid according to dosing intervals [18].

A lower dose of tedizolid, 200 mg once daily versus 600 mg twice daily of linezolid, may also contribute to a lower frequency or severity of reported side effects, especially if they are serum concentration dependent [19, 20]. Despite the two- to four-fold lower MIC_50_ and MIC_90_ for tedizolid, the use of the lower dose of tedizolid, a sixth of the daily dose of linezolid, needs to be evaluated for severe infections and especially during long-term treatment. A study evaluating the pharmacokinetics and response to exposure to tedizolid [19] showed that the incidence of treatment-emergent side effects increased with increased exposure to the drug. However, no such relationship was evident regarding hematologic side effects and exposure to tedizolid in that study. On the contrary, a recently published study [20] found alterations in haematological parameters, especially a decrease in mean platelet count, in a dose-dependent manner during 21-day courses of tedizolid.

The safety, tolerability, and adverse effects of long-term tedizolid treatment remain to be evaluated. However, tedizolid may represent an oxazolidinone that is better accepted by patients for long-term treatment, due to its favourable adverse effect profile and its once-a-day administration regime. Alone or in combination with rifampicin, tedizolid could be a possible treatment option in the management of PJIs caused by MDR *S. epidermidis* in both prosthesis retention following debridement and two-stage exchange surgery.
